# *rac*-3,3,3-Trifluoro­lactic acid

**DOI:** 10.1107/S1600536813003097

**Published:** 2013-02-02

**Authors:** Thomas Gerber, Richard Betz

**Affiliations:** a Nelson Mandela Metropolitan University, Summerstrand Campus, Department of Chemistry, University Way, Summerstrand, PO Box 77000, Port Elizabeth, 6031, South Africa

## Abstract

The title compound (systematic name: *rac*-3,3,3-trifluoro-2-hy­droxy­propanoic acid), C_3_H_3_F_3_O_3_, is a fluorinated derivative of lactic acid. The O=C—C—O(H) torsion angle is 13.26 (15)°. In the crystal, O—H⋯O hydrogen bonds and C—H⋯O contacts connect the mol­ecules into sheets perpendicular to the *c* axis.

## Related literature

For the crystal structure of 2-hy­droxy-2-(trifluoro­meth­yl)proprionic acid, see: Soloshonok *et al.* (2007 ▶). For background to chelate ligands, see: Gade (1998 ▶). For graph-set analysis of hydrogen bonds, see: Etter *et al.* (1990 ▶); Bernstein *et al.* (1995 ▶).
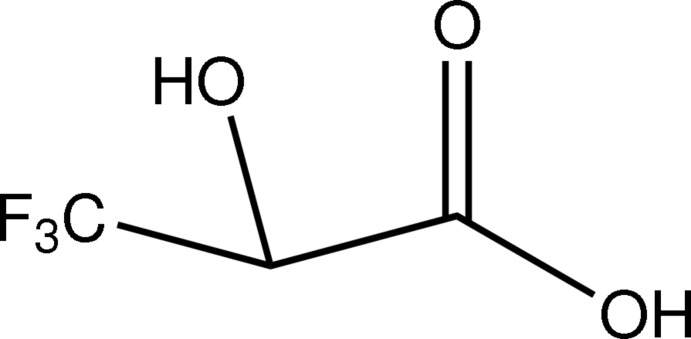


## Experimental

### 

#### Crystal data

C_3_H_3_F_3_O_3_
*M* *_r_* = 144.05
Orthorhombic, 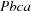

*a* = 10.586 (3) Å
*b* = 9.248 (3) Å
*c* = 10.826 (3) Å
*V* = 1059.9 (5) Å^3^
*Z* = 8
Mo *K*α radiation
μ = 0.22 mm^−1^
*T* = 200 K
0.40 × 0.30 × 0.25 mm

#### Data collection

Bruker APEXII CCD diffractometer
Absorption correction: multi-scan (*SADABS*; Bruker, 2008 ▶) *T*_min_ = 0.892, *T*_max_ = 1.000
9450 measured reflections
1309 independent reflections
1133 reflections with *I* > 2σ(*I*)
*R*_int_ = 0.017

#### Refinement

*R*[*F* ^2^ > 2σ(*F* ^2^)] = 0.031
*wR*(*F* ^2^) = 0.081
*S* = 1.06
1309 reflections
82 parameters
H-atom parameters constrained
Δρ_max_ = 0.31 e Å^−3^
Δρ_min_ = −0.19 e Å^−3^

### 

Data collection: *APEX2* (Bruker, 2010 ▶); cell refinement: *SAINT* (Bruker, 2010 ▶); data reduction: *SAINT*; program(s) used to solve structure: *SHELXS97* (Sheldrick, 2008 ▶); program(s) used to refine structure: *SHELXL97* (Sheldrick, 2008 ▶); molecular graphics: *ORTEP-3 for Windows* (Farrugia, 2012 ▶) and *Mercury* (Macrae *et al.*, 2008 ▶); software used to prepare material for publication: *SHELXL97* and *PLATON* (Spek, 2009 ▶).

## Supplementary Material

Crystal structure: contains datablock(s) I, global. DOI: 10.1107/S1600536813003097/zl2532sup1.cif

Supplementary material file. DOI: 10.1107/S1600536813003097/zl2532Isup2.cdx

Structure factors: contains datablock(s) I. DOI: 10.1107/S1600536813003097/zl2532Isup3.hkl

Supplementary material file. DOI: 10.1107/S1600536813003097/zl2532Isup4.cml

Additional supplementary materials:  crystallographic information; 3D view; checkCIF report

## Figures and Tables

**Table 1:** Hydrogen-bond geometry (Å, °)

*D*—H⋯*A*	*D*—H	H⋯*A*	*D*⋯*A*	*D*—H⋯*A*
O3—H12⋯O2^i^	0.84	2.03	2.7459 (13)	143
O1—H111⋯O3^ii^	0.84	1.80	2.6381 (13)	172
C2—H12*A*⋯O1^iii^	1.00	2.60	3.4588 (16)	144

Symmetry codes: (i) 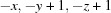; (ii) 
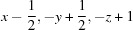
; (iii) 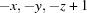.

**Fig. 1.**
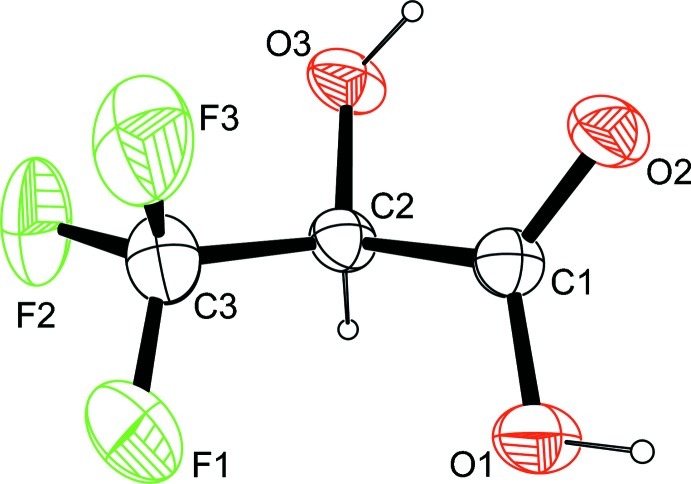
The molecular structure of the title compound, with atom labels and anisotropic displacement ellipsoids (drawn at 50% probability level).

**Fig. 2.**
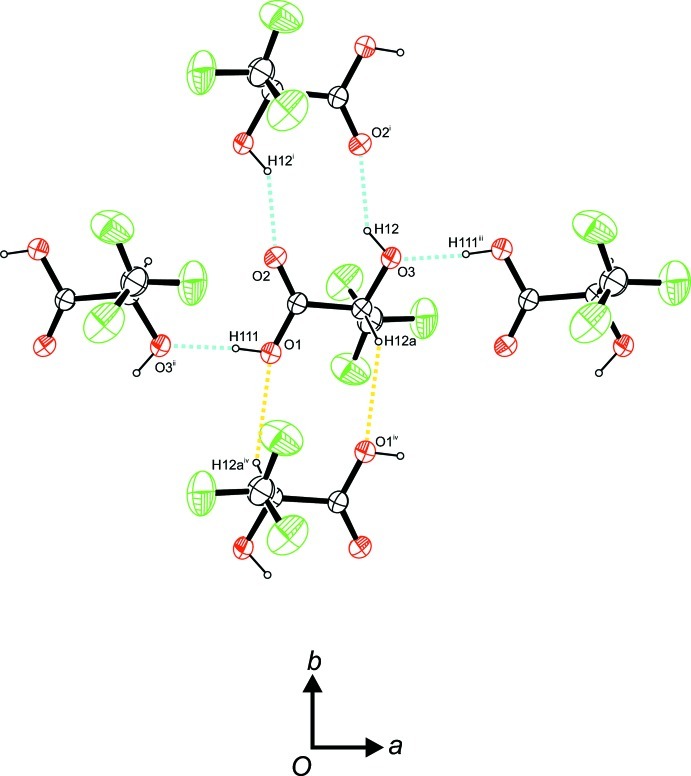
Intermolecular contacts, viewed along [0 0 - 1]. Blue dashed lines indicate hydrogen bonds, yellow dashed lines indicate C–H···O contacts. Symmetry operators: i -x, -y + 1, -z + 1; iix - 1/2, -y + 1/2, -z + 1; iiix + 1/2, -y + 1/2, -z + 1; iv -x, -y, -z + 1.

**Fig. 3.**
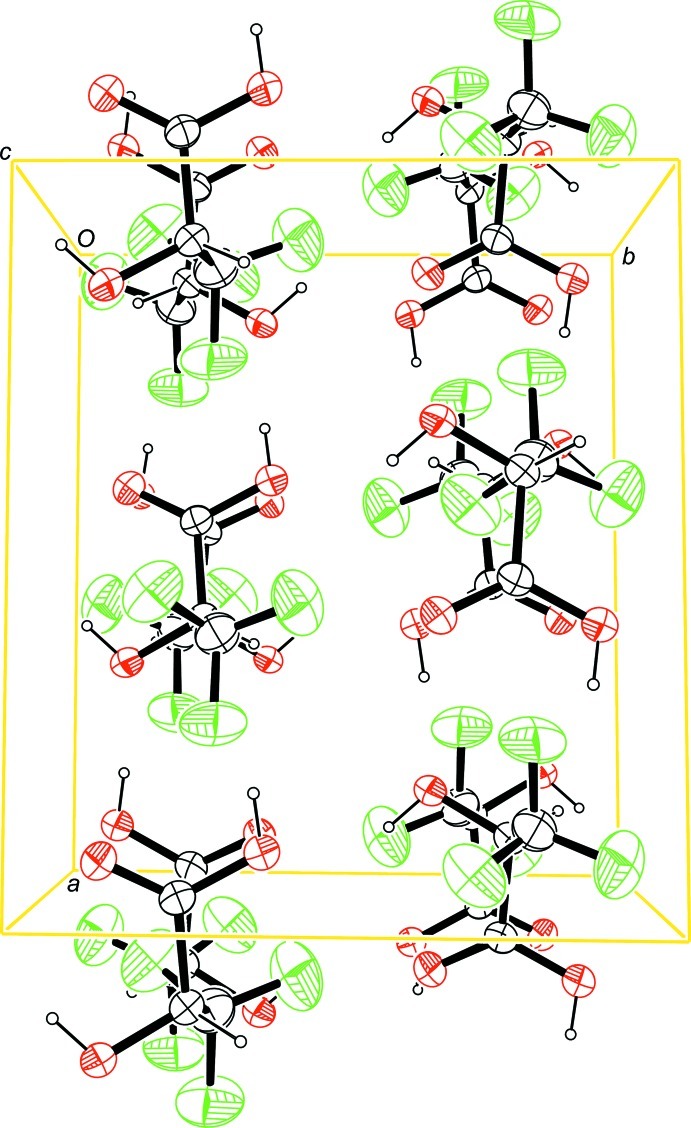
Molecular packing of the title compound, viewed along [0 0 - 1] (anisotropic displacement ellipsoids drawn at 50% probability level).

## supplementary crystallographic information

### Comment

Chelate ligands have found widespread use in coordination chemistry due to the increased stability of coordination compounds they can form in comparison to monodentate ligands (Gade, 1998). Hydroxycarboxylic acids are particularily interesting in this aspect as they offer two hydroxyl groups of markedly different acidity as potential bonding partners. Upon varying the substitution pattern on the hydrocarbon backbone, the acidity of the respective hydroxyl groups can be finetuned over a wide range and they may, thus, serve as probes for establishing the rules in which pK_a_ range coordination to various central atoms can be observed. To allow for comparisons of metrical parameters of the carboxylic-acid-derived ligand in envisioned coordination compounds, the crystal and molecular structure of 3,3,3-trifluorolactic acid as the free ligand was determined. The crystal structure of a related compound, 2-hydroxy-2-(trifluoromethyl)proprionic acid, is apparent in the literature (Soloshonok *et al.*, 2007).

The carboxyl group is nearly in plane with the C–OH moiety. The respective O═C–C–O(H) dihedral angle was found at 13.26 (15) ° only (Fig. 1).

In the crystal, intra- as well as intermolecular hydrogen bonds are apparent. The former ones appear between the alcoholic hydroxyl group as donor and the ketonic oxygen atom as acceptor and, therefore, might be the cause for the small dihedral angle discussed above. The intermolecular hydrogen bonds are supported by the carboxyl group as donor and the alcoholic hydroxyl group as acceptor. In addition, C–H···O contacts whose range falls by more than 0.1 Å below the sum of van-der-Waals radii of the corresponding atoms can be observed. These stem from the hydrogen atom of the methine group and apply the oxygen atom of the carboxylic hydroxyl group as acceptor. Metrical parameters as well as information about the symmetry of these hydrogen bonds is summarized in Table 1. In terms of graph-set analysis (Etter *et al.*, 1990; Bernstein *et al.*, 1995), the descriptors for the hydrogen bonds are *C*^1^_1_(5) and *R*^2^_2_(10) on the unary level while the C–H···O contacts necessitate a *R*^2^_2_(8) descriptor on the same level. In total, the intermolecular interactions connect the molecules to planes perpendicular to the crystallographic *c* axis (Fig. 2).

The packing of the title compound in the crystal structure is shown in Figure 3.

### Experimental

The compound was obtained from Alfa Aesar. Crystals suitable for the diffraction study were taken directly from the provided product.

### Refinement

The carbon-bound H atom of the methine group was placed in a calculated position (C–H 1.00 Å) and was included in the refinement in the riding model approximation, with *U*(H) set to 1.2*U*_eq_(C). The H atoms of the hydroxyl groups were allowed to rotate with a fixed angle around the C–O bond to best fit the experimental electron density (HFIX 147 in the *SHELX* program suite (Sheldrick, 2008)), with *U*(H) set to 1.5*U*_eq_(O).

### Crystal data

**Table:**

C_3_H_3_F_3_O_3_	*F*(000) = 576
*M**_r_* = 144.05	*D*_x_ = 1.806 Mg m^−^^3^
Orthorhombic, *P**b**c**a*	Mo *K*α radiation, λ = 0.71073 Å
Hall symbol: -P 2ac 2ab	Cell parameters from 4117 reflections
*a* = 10.586 (3) Å	θ = 3.5–28.2°
*b* = 9.248 (3) Å	µ = 0.22 mm^−^^1^
*c* = 10.826 (3) Å	*T* = 200 K
*V* = 1059.9 (5) Å^3^	Platelet, colourless
*Z* = 8	0.40 × 0.30 × 0.25 mm

### Data collection

**Table:**

Bruker APEXII CCD diffractometer	1309 independent reflections
Radiation source: fine-focus sealed tube	1133 reflections with *I* > 2σ(*I*)
Graphite monochromator	*R*_int_ = 0.017
φ and ω scans	θ_max_ = 28.3°, θ_min_ = 3.5°
Absorption correction: multi-scan (*SADABS*; Bruker, 2008)	*h* = −14→10
*T*_min_ = 0.892, *T*_max_ = 1.000	*k* = −12→12
9450 measured reflections	*l* = −14→14

### Refinement

**Table:**

Refinement on *F*^2^	Primary atom site location: structure-invariant direct methods
Least-squares matrix: full	Secondary atom site location: difference Fourier map
*R*[*F*^2^ > 2σ(*F*^2^)] = 0.031	Hydrogen site location: inferred from neighbouring sites
*wR*(*F*^2^) = 0.081	H-atom parameters constrained
*S* = 1.06	*w* = 1/[σ^2^(*F*_o_^2^) + (0.0349*P*)^2^ + 0.3452*P*] where *P* = (*F*_o_^2^ + 2*F*_c_^2^)/3
1309 reflections	(Δ/σ)_max_ < 0.001
82 parameters	Δρ_max_ = 0.31 e Å^−^^3^
0 restraints	Δρ_min_ = −0.19 e Å^−^^3^

### Fractional atomic coordinates and isotropic or equivalent isotropic displacement parameters (Å^2^)

**Table:**

	*x*	*y*	*z*	*U*_iso_*/*U*_eq_	
F1	0.07491 (11)	0.07334 (11)	0.26362 (9)	0.0698 (3)	
F2	0.24046 (9)	0.20543 (13)	0.26938 (9)	0.0670 (3)	
F3	0.06121 (11)	0.29936 (12)	0.22550 (8)	0.0661 (3)	
O3	0.16058 (7)	0.35907 (8)	0.46617 (9)	0.0346 (2)	
H12	0.1084	0.4270	0.4731	0.052*	
O1	−0.10270 (7)	0.12077 (8)	0.45966 (9)	0.0361 (2)	
H111	−0.1790	0.1327	0.4782	0.054*	
O2	−0.09203 (7)	0.35985 (8)	0.48706 (9)	0.0348 (2)	
C1	−0.04410 (10)	0.24515 (11)	0.46318 (10)	0.0256 (2)	
C2	0.09691 (10)	0.23181 (11)	0.43390 (10)	0.0260 (2)	
H12A	0.1328	0.1499	0.4829	0.031*	
C3	0.11835 (13)	0.20173 (15)	0.29671 (12)	0.0398 (3)	

### Atomic displacement parameters (Å^2^)

**Table:**

	*U*^11^	*U*^22^	*U*^33^	*U*^12^	*U*^13^	*U*^23^
F1	0.0956 (8)	0.0594 (6)	0.0543 (6)	−0.0146 (5)	0.0143 (5)	−0.0292 (5)
F2	0.0451 (5)	0.1003 (8)	0.0556 (5)	0.0055 (5)	0.0226 (4)	−0.0110 (5)
F3	0.0827 (7)	0.0809 (7)	0.0348 (5)	0.0224 (6)	0.0003 (4)	0.0123 (4)
O3	0.0212 (4)	0.0258 (4)	0.0568 (5)	0.0024 (3)	−0.0003 (3)	−0.0067 (4)
O1	0.0232 (4)	0.0251 (4)	0.0600 (6)	0.0008 (3)	0.0000 (4)	−0.0019 (4)
O2	0.0271 (4)	0.0252 (4)	0.0523 (5)	0.0034 (3)	0.0029 (4)	−0.0034 (3)
C1	0.0227 (5)	0.0257 (5)	0.0285 (5)	0.0020 (4)	−0.0023 (4)	0.0008 (4)
C2	0.0229 (5)	0.0237 (5)	0.0315 (5)	0.0026 (4)	−0.0003 (4)	−0.0006 (4)
C3	0.0393 (7)	0.0437 (7)	0.0362 (6)	0.0038 (5)	0.0053 (5)	−0.0030 (5)

### Geometric parameters (Å, º)

**Table:**

F1—C3	1.3227 (17)	O1—H111	0.8399
F2—C3	1.3265 (17)	O2—C1	1.2040 (13)
F3—C3	1.3325 (17)	C1—C2	1.5310 (15)
O3—C2	1.4005 (13)	C2—C3	1.5280 (17)
O3—H12	0.8400	C2—H12A	1.0000
O1—C1	1.3074 (13)		
			
C2—O3—H12	109.5	C3—C2—H12A	108.8
C1—O1—H111	109.5	C1—C2—H12A	108.8
O2—C1—O1	125.56 (10)	F1—C3—F2	107.55 (12)
O2—C1—C2	121.76 (10)	F1—C3—F3	107.08 (12)
O1—C1—C2	112.68 (9)	F2—C3—F3	107.21 (12)
O3—C2—C3	108.91 (10)	F1—C3—C2	112.03 (11)
O3—C2—C1	110.48 (8)	F2—C3—C2	110.91 (11)
C3—C2—C1	111.15 (9)	F3—C3—C2	111.81 (11)
O3—C2—H12A	108.8		
			
O2—C1—C2—O3	13.26 (15)	C1—C2—C3—F1	−66.42 (14)
O1—C1—C2—O3	−166.37 (9)	O3—C2—C3—F2	51.46 (14)
O2—C1—C2—C3	−107.76 (12)	C1—C2—C3—F2	173.40 (11)
O1—C1—C2—C3	72.61 (12)	O3—C2—C3—F3	−68.14 (13)
O3—C2—C3—F1	171.65 (10)	C1—C2—C3—F3	53.80 (14)

### Hydrogen-bond geometry (Å, º)

**Table:**

*D*—H···*A*	*D*—H	H···*A*	*D*···*A*	*D*—H···*A*
O3—H12···O2^i^	0.84	2.03	2.7459 (13)	143
O1—H111···O3^ii^	0.84	1.80	2.6381 (13)	172
C2—H12*A*···O1^iii^	1.00	2.60	3.4588 (16)	144

Symmetry codes: (i) −*x*, −*y*+1, −*z*+1; (ii) *x*−1/2, −*y*+1/2, −*z*+1; (iii) −*x*, −*y*, −*z*+1.
